# Corporation Monopolies in Ireland

**Published:** 1829-07-01

**Authors:** 


### XXII.

### CORPORATION MONOPOLIES—MAMMON OPENLY ADORED—PATRIOTISM OF THE IRISH APOTHECARIES' COMPANY.

It is fortunate that vicious principles generally work their own destruction—and that the attempt to enforce old, obsolete, and unjust statutes, (unjust as applicable to times present,) ends in the abrogation of those statutes, and the enactment of regulations somewhat in consonance with the advance of knowledge and the spirit of the age in which we live. Ireland seems destined to afford striking illustrations of these remarks. The adamantine chains of bigotry and intolerance by which five millions of her inhabitants have been, for centuries, held in disgraceful thraldom, are rent asunder by the voice of an enlightened legislature—but not before gigantic efforts were made by those who were oppressed. With the hope, no doubt, of imitating their superiors, the Apothecaries' Company in Dublin, appear determined to shew their "brief authority" to the utmost stretch of the letter of the law—especially as the exercise of their prerogative has the double effect of legally picking the pockets of others, and replenishing their own! Cupid is painted blind—and so should cupidity. The little tyrants of the pestle and mortar have over-reached the mark; and by the attempt to place on a level with quacks and impostors those men who have graduated in regular universities—who have served their king and country by "flood and field"—and who are consequently superior to themselves —by this base attempt, we say, they have roused the spirit of the injured party, and a forcible appeal is made to Parliament, which is likely to end in a termination of their monopoly.

Some forty years ago the wisdom of our ancestors, in the Sister Kingdom, enacted a law, that all young gentlemen designed for the medical profession, should undergo an examination as to their classical knowledge previous to apprenticeship, and another examination, as to medical and pharmaceutical acquirements, before embarking in the practice of their profession. We do not find fault with the act, but with those who are carrying the act into operation. Whatever may have been the words, it is clear that it could not have been the object of the bill to prevent other than ignorant or unqualified persons from practising as apothecaries. It would be absurd to suppose that it was meant to prohibit physicians from dispensing their own medicines if they thought proper! Is it not odious and disgusting that the penalty of one hundred pounds which was ordered to be levied on quacks and pretenders who should presume to practise without education or qualification, should now be levied on physicians and surgeons who have all passed a *much higher tribunal* than that before which they are summoned! That the whole and sole object of this movement among the jalapmongers of Dublin, is sordid pelf, will be patent as the sun in the firmament, by the following observations of under-secretary Dawson in the House of Commons:—

"Mr. Dawson had two similar petitions to present, one from the physicians, surgeons, and apothecaries of the county of Londonderry; the other from the same description of medical practitioners in the county of Tyrone. The petitioners complained of the peculiar hardship to which they were exposed, by means of the monopoly enjoyed by the Apothecaries' Company. The act of 1791, by which the Apothecaries' Hall in Dublin was incorporated, prevented individuals from compounding and dispensing medicines who had not received a licence from the company. The object of the restriction might have been a very wise one; it was probably intended by these means to hinder unskilful persons from compounding drugs. But the petitioners did not come under that denomination. The act already referred to contained a clause, authorizing the Apothecaries' Hall to recover a penalty of 100*l.* from every person who should attempt to exercise the business of an apothecary without their licence. The consequence of the way in which this penalty was now imposed, was to render an act passed to protect the health of His Majesty's subjects the means of filling the pockets of the Apothecaries' Company. The Apothecaries' Hall had commenced actions against many individuals for compounding and dispensing medicines without having complied with the regulations of the company. In adopting this course they had not exhibited the slightest inclination to serve the public, by preventing incompetent persons from acting as apothecaries: their sole object appeared to be to minister to their own profit by securing the penalties. They had chosen skilful and able persons against whom to enforce the law, practitioners who, having received their diplomas from the colleges of London, Glasgow, Edinburgh, and Dublin, thought it derogatory to their dignity to go to the Apothecaries' Hall for the purpose of taking out a licence. These persons had been selected as victims, and the worst consequences must ensue if a stop were not put to the mischief. There were not fewer than 256 medical practitioners, who had taken out their diplomas in the manner described, and who were now resident in the north of Ireland, where they exercised the profession of physicians, surgeons, and apothecaries, rendering themselves, by their necessary assumption of the last-named calling, liable to the penalties which it was in the power of the Apothecaries' Company to inflict. It was said to be the object of the Apothecaries' Hall in Dublin, to put these 250

gentlemen under an annual fine or mulct of 20*l.*—the sum demanded for a licence; and thus to acquire a revenue of 5,000*l.* a year at their expense. What the Apothecaries' Company aimed at was, not to prevent unskilful persons from practising as apothecaries, but to benefit themselves in a pecuniary point of view, and keep up their monopoly. They never inquired if a man were skilful or not, but whether he was able to pay the penalty. When he paid it, they sent him a certificate of practice without any examination. It appeared perfectly ridiculous that persons capable of acting as physicians, surgeons, and apothecaries in the army and navy—who had retired on half-pay, and who were sufficiently skilful and intelligent in their profession to be of the utmost service to the community—should not be qualified to act as apothecaries without licence from a body of men such as the Apothecaries' Company. Yet that was the situation in which 250 practitioners in the north of Ireland were placed, and from which they prayed to be relieved. The petitioners deprecated the monopoly, and the unjust taxation imposed by the Apothecaries' Hall. He called the attention of his noble friend (the Chief Secretary of State for Ireland) to the subject, which he conceived to be one that could not be passed over without investigation. The petitioners prayed for relief from the operation of the act of 1791, on their own behalf, and on that of all persons who had obtained licences or degrees from any of the royal medical colleges in Great Britain or Ireland. That did not appear an unreasonable proposition: a diploma thus obtained was a better security than a certificate of the Apothecaries' Company. He hoped his noble friend would bring in a bill without delay to remove the inconvenience of the present system. The Apothecaries' Company were so well aware that the subject was one which would never stand the test of inquiry, that they were giving orders to levy the fines with the utmost expedition against the gentlemen who had rendered themselves liable to them. No time should be lost in taking the matter up; the subject was one which called for the interposition of the house, not only for the sake of the medical profession, but for the sake of the inhabitants of the country, more particularly of the north of Ireland."

"Lord F. L. Gower assured his hon. friend, who had appealed personally to him on the subject, that he had not overlooked the matter to which his honourable friend now directed his attention. Upon the presentation of a petition from the county of Down, on a former evening, to the same effect as those now presented, he took an opportunity of expressing his concurrence in the object of it as far as he could do without prejudging the case. He had put himself in communication with the authorities on the other side of the water, for the purpose of ascertaining whether any answer could be made by the Apothecaries' Company to the allegations of the petitioners. He did not yet know the nature of the representations that would be made by the company; but he wished, as much as any man, that the evil, if it were of the kind, or to any thing like the extent stated, should be remedied. He believed, that under the statute incorporating the Apothecaries' Hall, any practising apothecary might become a member of the company on the payment of 100*l.* He was not aware that the company had adhered very strictly to the provisions of the statute, or exercised a prudent discretion in the course they had adopted.

"The petitions were ordered to be printed."

The knights of the pestle in Dublin will not be slow to perceive that these golden visions of five thousand a year from the unlicensed graduates in the North, are likely to be dissolved into thin air by Lord Gower and a British Parliament—not over nice in questioning the "wisdom of our ancestors," or even revising statutes that were, like the laws of the Medes and Persians, to have been irrevocable.

We hope that the words of Lord Gower and Mr. Dawson will not be entirely lost on certain corporate bodies—and especially the Company of Apothecaries, on this side of the Channel.

The very same vexatious, and, we would add, unjust regulations exist in this country. If Sir Henry Halford, Sir Astley Cooper, or Sir Gilbert Blane, were, from choice or necessity, determined to add pharmacy to their other avocations, they must present themselves for examination and a licence at "THE HALL!!" So if a man have served in the highest offices of the army or navy medical departments—or have graduated at the first universities of Europe, he must have the licence of "THE HALL," before he can administer a saline draught, or a dose of calomel! We do not know whether or not the Apothecaries' Company insist on an actual examination in these cases—but if they demand any fee for the licence, we maintain that they act illiberally and unjustly. All they should require is a sight of the diploma from a regular university—or the medical commission in army or navy. Those documents produced, the licence should be granted—or rather the declaration should be made that the individuals alluded to did not come within the virtual meaning of the act of 1815. This would be the just, liberal, and honorable procedure—and if a contrary system be pursued, the aggrieved party should petition Parliament, as has been done by their brethren in the North of Ireland.

Sat verbum sapientibus.

---

